# Yeast two‐ and three‐species hybrids and high‐sugar fermentation

**DOI:** 10.1111/1751-7915.13390

**Published:** 2019-03-05

**Authors:** Matthias Sipiczki

**Affiliations:** ^1^ Department of Genetics and Applied Microbiology University of Debrecen Debrecen Hungary

## Abstract

The dominating strains of most sugar‐based natural and industrial fermentations either belong to *Saccharomyces cerevisiae* and *Saccharomyces uvarum* or are their chimeric derivatives. Osmotolerance is an essential trait of these strains for industrial applications in which typically high concentrations of sugars are used. As the ability of the cells to cope with the hyperosmotic stress is under polygenic control, significant improvement can be expected from concerted modification of the activity of multiple genes or from creating new genomes harbouring positive alleles of strains of two or more species. In this review, the application of the methods of intergeneric and interspecies hybridization to fitness improvement of strains used under high‐sugar fermentation conditions is discussed. By protoplast fusion and heterospecific mating, hybrids can be obtained that outperform the parental strains in certain technological parameters including osmotolerance. Spontaneous postzygotic genome evolution during mitotic propagation (GARMi) and meiosis after the breakdown of the sterility barrier by loss of *MAT* heterozygosity (GARMe) can be exploited for further improvement. Both processes result in derivatives of chimeric genomes, some of which can be superior both to the parental strains and to the hybrid. Three‐species hybridization represents further perspectives.

## Introduction

Yeasts play an essential role in bread‐making, the fermentation of alcoholic beverages and the production of bioethanol. Although a high number of fermentative yeast species have biotechnological relevance, the strains dominating most natural and industrial fermentations either belong to *S. cerevisiae* and *S. uvarum* or are their chimeric derivatives including the genetically highly diverse ‘hybrid species’ *S*. *pastorianus/carlsbergensis* and *S. bayanus*. During the fermentation of high‐sugar and high‐gravity substrates, the *Saccharomyces* cells are exposed to hyperosmotic stress. To counterbalance the extracellular solute concentrations, the cells accumulate glycerol. Since the response to the hyperosmotic shock is a polygenic trait, significant improvement can be expected from concerted modification of the activity of multiple genes rather than from the manipulation of individual genes (e.g. Albertyn *et al*., 1994; Shi *et al*., 2018). Hybridization brings all alleles of all relevant genes of different strains together and recombine them during segregation/chimerization of the hybrid genomes. The hybrids and/or their chimeric derivatives can outperform the parental strains in certain technologically relevant properties including stress response. In this review, I describe basic knowledge about the hyperosmotic stress response and the exploitation of interspecies hybridization and hybrid evolution in improving the fitness of the strains under high‐sugar conditions.

## Yeasts face hyperosmotic stress during fermentation

The yeast cells have to cope with high‐sugar and/or VHG (very high gravity) conditions in the fermentation of certain types of beverages, VHG brewing and VHG fuel ethanol production technologies, fermented vegetable extracts and high‐sugar dough. When preparing dessert wines from late‐harvest or botrytized grapes, the sugar concentrations in the must can be as high as 60% (e.g. Donéche, 1993; Sipiczki *et al*., 2010). In ice‐wine grape juice, the sugar content can reach 50% (Erasmus *et al*., 2004). The total sugar content of sugar cane and sugar beet molasses fermented in traditional alcohol distilleries varies between 40% and 50% (e.g. Doelle and Docile, 1990; Khoja *et al*., 2018). Sweet dough (high‐sugar dough) contains up to approximately 30% sucrose (Sasano *et al*., 2012), and high‐sugar vegetable extracts can have 40–60% sugar (Ok and Hashinaga, 1997). In VHG brewing, the gravity of the wort can exceed 20° P (Huuskonen *et al*., 2010). In VHG fuel ethanol fermentation, the mashes contain sugar at concentrations higher than 25% to achieve ethanol yields higher than 15%. Certain *S. cerevisiae* strains can grow and vigorously ferment potato mash containing as much as 40% (2.2 M) glucose (Watanabe *et al*., 2010). But not all *Saccharomyces* strains can cope with sugar added at such high concentration to the medium and strains of other genera can be more tolerant.

## The effect of hyperosmotic stress on the *Saccharomyces* cells

Upon being exposed to hyperosmotic conditions, yeast cells cease growing and propagating, rapidly lose intracellular water, thereby resulting in a loss of turgor pressure followed by shrinkage of the cytoplasm (Slaninova *et al*., 2000; Schaber and Klipp, 2008; Munna *et al*., 2015). In the shrinking cytoplasm, the microtubular and actin‐based cytoskeletal structures become disorganized and deep plasma membrane invaginations are formed which the cells can fill up with amorphous cell wall material (Chowdhury *et al*., 1992; Slaninova *et al*., 2000). The overall cell volume is also reduced, and the yeast cells have to adapt their internal osmolarity to the hyperosmotic external conditions to restore the optimal cell volume (e.g. Pratt *et al*., 2003; Munna *et al*., 2015; Talemi *et al*., 2016).

## The complexity of the response of the *Saccharomyces* cells to hyperosmotic stress

**Figure 1:**
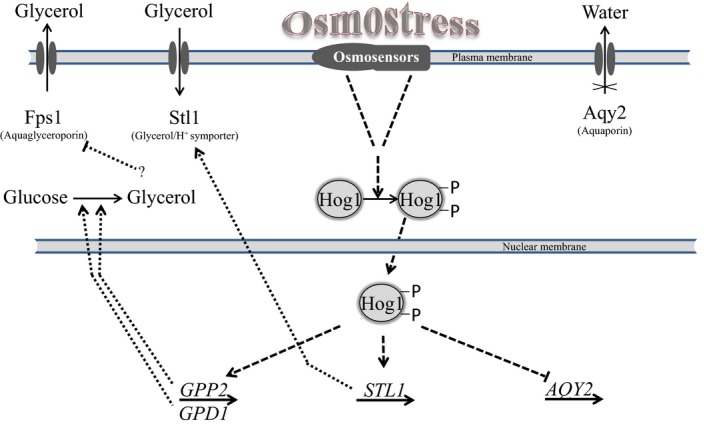
A schematic diagram of the roles of the Hog1 protein kinase phosphorylated by the HOG MAPK (Mitogen‐Activated Protein Kinase) pathway in the response of the *Saccharomyces* cell to the osmotic stress. The diagram is based on the review of Hohmann (2015).

To cope with an increased external osmolarity caused by the high sugar concentration in the environment, the *Saccharomyces* cells initiate a complex adaptive program that includes the synthesis and retention of the compatible osmolyte glycerol, temporary arrest of cell‐cycle progression, altered transcription and translation patterns. The genes and processes involved in the response have been reviewed in several recent studies (Saito and Posas, 2012; Hohmann, 2015; Saxena and Sitaraman, 2016; Taymaz‐Nikerel *et al*., 2016; Auesukaree, 2017) and can be summarized as follows (Fig. 1).

The response is basically governed by the high‐osmolarity glycerol (HOG) signalling pathway, whose core is the Hog1 MAP kinase (MAPK) cascade. The phosphorylated Hog1 enters the nucleus and activates the expression of several transcription factors, each of which is responsible for controlling the expression of a subset of osmoresponsive genes. Due to the activity of these genes, the intracellular level of glycerol increases which prevents the efflux of water from the cell into the environment. *STL1* codes for a H^+^ symporter that transports glycerol inside the cell. *GPD1* encodes a NADH‐dependent glycerol‐3‐phosphate dehydrogenase that reduces dihydroxy‐acetone‐phosphate to glycerol‐3‐phosphate. Glycerol‐3‐phosphate is further converted to glycerol by the glycerol‐3‐phosphate phosphatase encoded by *GPP2*. In response to osmostress, the glycerol efflux mediated by the Fps1 aquaglyceroporin closes to keep the glycerol inside the cell, but this effect seems to be independent of Hog1. Hog1 was found to down‐regulate the expression of the aquaporin Aqy2 and thereby restrict water loss. Apart from the genes directly involved in the HOG cascade, large numbers of additional genes have been implicated in the response by transcriptomic analyses and testing of deletion mutants (e.g. Erasmus *et al*., 2003; O'Rourke and Herskowitz, 2004; Ando *et al*., 2006; Tanaka‐Tsuno *et al*., 2007; Jimenez‐Marti *et al*., 2011). Recent studies reported results demonstrating that mitochondrial activity is also required for proper osmotic stress adaptation (Pastor *et al*., 2009; Gonzalez *et al*., 2016). The high number of up‐ and down‐regulated genes indicates that a large proportion of the gene pool of the yeast cell has to be fine‐tuned to give an adequate response to the high external osmolarity.

## Correlation between the presence of foreign genes in the genome in ‘natural’ strains and their stress response

In numerous wine and industrial strains isolated from natural fermenting yeast communities, correlation was observed between the presence of genes of two or even more *Saccharomyces* species and the more adequate response of their cells to certain types of stress (for a review, see Marsit and Dequin, 2015). For example, *S. cerevisiae* strains harbouring genes from the more cryotolerant species *S*. *uvarum* or *S. kudriavzevii* are better adapted to growth and fermentation at low temperatures than the strains of ‘pure’ *S. cerevisiae* genomes (e.g. Peris *et al*., 2018). The proportion of the non‐*cerevisiae* gene pool varies in these chimeric strains from a few genes to an (almost) complete subgenome (e.g. Erny *et al*., 2012; Peris *et al*., 2018). The transgressive stress‐response phenotypes of certain natural chimeric strains indicate that stress tolerance might be improved synthetically by admixing genes of species. Examples of modification of osmotolerance by interspecies hybridization and posthybridization (postzygotic) genome evolution are shown in Table 1.

**Table 1:** Examples of modulation of osmotolerance by hybridization and hybrid segregation

Species combination	New phenotype	Reference
Somatic hybridization (protoplast fusion)		
*S. cerevisiae* × *Z. mellis*	Efficient fermentation at to 30% glucose combined with good yields of ethanol production	Legmann and Margalith (1983, 1986)
*S. cerevisiae* × *D. hansenii*	Combination of the higher thermotolerance and growth rate of the *S. cerevisiae* parent with higher osmotolerance from the *D. hansenii* parent	Loray *et al*. (1995)
*S. cerevisiae* × *T. delbrueckii*	Tolerance to increased glucose concentrations (up to 70%) combined with increased arabinitol and glycerol production	Lucca *et al*. (1999, 2002)
*S. diastaticus* (*S. cerevisiae* var. *diastaticus*) × *Z. rouxii*	Fermentation and sugar utilization patterns of *S. diastaticus* combined with increased osmotolerance	Spencer *et al*. (1985)
*S. diastaticus* (*S. cerevisiae* var. *diastaticus*) × *S. uvarum*	Fermentation rate superior to those of the parental strain at 30% sugar combined with the ability to ferment at 40 °C	Stewart *et al*. (1988)
Sexual hybridization (mating, conjugation)		
*S. cerevisiae* × *S*. *carlsbergensis*	Improved fermentation rates in high‐gravity wort (18° Plato) relative to the ale parent	Garcia Sanchez *et al*. (2012)
*S. cerevisiae* × *S. eubayanus*	Increased fermentation rate in very high‐gravity wort (25° Plato) relative to the parent strains	Krogerus *et al*. (2016)
*S. cerevisiae* × *S. kudriavzevii*	Increased efficiency of fructose and glucose consumption in high‐sugar wine fermentations	Lopandic *et al*. (2016); Gangl *et al*. (2017)
*S*. *cerevisiae* × *S. mikatae*	Combination of low‐temperature tolerance (from *S. mikatae*) with high‐temperature tolerance and osmotolerance (from *S. cerevisiae*)	Bellon *et al*. (2013)
*S. cervisiae* × *S. uvarum*	Increased fermentation efficiency, ethanol and glycerol production in most of 32% sugar	Restuccia *et al*. (2011)
*S. cerevisiae* × *S. uvarum*	Slightly increased osmotolerance in certain hybrids	Pfliegler *et al*. (2014)
*S. cervisiae* × *S. uvarum*	Combination of fermentation phenotypes from both parents: robust fermentation in high‐sugar juice (25% and 35.5%) and the production of wines with low volatile acidity	Bellon *et al*. (2015)
Hybrid segregation by GARMi		
*S. cerevisiae* × *S. uvarum*	A segregant becoming dominant after five rounds of fermentation of high‐sugar grape must (evolved hybrid) fermented the grape juice of 35% sugar at much faster rate than the original hybrid strain	Bellon *et al*. (2018)
Hybrid segregation by GARMe		
*S. cerevisiae* × *S. kudriavzevii*	Increased efficiency of fructose and glucose consumption in high sugar wine fermentations in certain F1 spore clones	Lopandic *et al*. (2016)
*S. cerevisiae* × *S*. *uvarum*	Slightly increased osmotolerance in certain F1 spore clones	Pfliegler *et al*. (2014)
*S. diastaticus* (*S. cerevisiae* var. *diastaticus*) × *Z. rouxii*	Certain spore clones had greater capacity to raise sweet dough than the hybrid	Spencer *et al*. (1985)

## Improving high‐sugar osmotolerance by interspecific and intergeneric hybridization

### Somatic hybridization (protoplast fusion)

**Figure 2:**
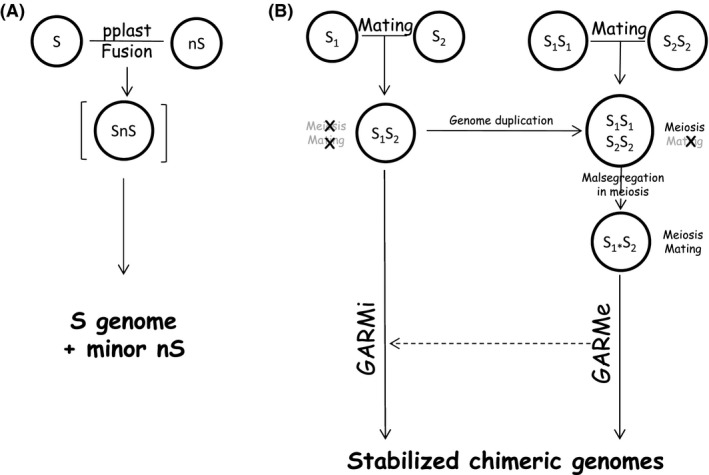
Interspecies hybridization and the evolution of the hybrid genome. A. Somatic hybridization (protoplast fusion). B. Hybridization by sexual conjugation (mating). NS: non‐*Saccharomyces* species; S: *Saccharomyces*; S_1_: *Saccharomyces* species 1; S_2_: *Saccharomyces* species 2. Star marks incomplete subgenome. GARMi: Genome Autoreduction in mitosis. GARMe: Genome Autoreduction in meiosis.

Osmotolerant somatic hybrids (growing at high sugar or salt concentrations) were obtained by protoplast fusion between *S. cerevisiae* and *Zygosaccharomyces mellis* (Legmann and Margalith, 1983, 1986), *S. diastaticus* (*S. cerevisiae* var. *diastaticus*) and *Z. rouxii* (Spencer *et al*., 1985), *S. diastaticus* (*S. cerevisiae* var. *diastaticus*) and *S. uvarum* (Stewart *et al*., 1988), *S. cerevisiae* and *Debaromyces hansenii* (Loray *et al*., 1995) and *S. cerevisiae* and *Torulaspora delbrueckii* (Lucca *et al*., 1999). The *S. diastaticus* × *Z. rouxii* hybrid was tolerant of sugar concentrations that were higher than those permitting the growth of the *S. diastaticus* parent. The viable spores of the hybrid formed clones of vegetative cells that had a much greater dough‐raising capacity than either the original hybrid or a commercial baker's yeast (Spencer *et al*., 1985). Unfortunately, little has been revealed from the genome structures of these hybrids. The few experimental data available indicate that the intergeneric hybrids had *Saccharomyces* genomes supplemented with only small percentages of the genomes of the non‐*Saccharomyces* fusion partners (Spencer *et al*., 1985; Salek, 2002) (Fig. 2A).

### Hybridization by mating (conjugation)

In the genus, *Saccharomyces* interspecies hybrids can be generated also by natural sexual hybridization because the reproductive isolation of the species is ‘postzygotic’. Cells of different *Saccharomyces* species can form viable hybrids by sexual mating (conjugation) but the hybrids are sterile. The sterility is mainly due to the failure of the (allosyndetic, homeologous) chromosomes to pair in meiosis (no functional gametes are produced) and to *MAT* heterozygosity (mating‐specific genes are repressed) (for a review, see Sipiczki, 2018). Each species can form sterile hybrids by mating with each other species, and the hybrids can have advantageous ‘transgressive’ traits in their phenotypes.

Hybrids of *S. cerevisiae* and *S. uvarum* strains were constructed that displayed combinations of positive phenotypic traits of the parents: robust fermentation in high‐sugar grape juice (from *S. cerevisiae*) and the production of wines with low volatile acidity (from *S. uvarum*) (Restuccia *et al*., 2011; Bellon *et al*., 2015). A different combination of strains of these species and the mating of *S. cerevisiae* × *S. kudriavzevii* resulted in hybrids showing improved fermentation rate in a must of high sugar content (Lopandic *et al*., 2016; Gangl *et al*., 2017). The *S. cerevisiae* × *S. paradoxus* and *S*. *cerevisiae* × *S. mikatae* hybrids inherited the osmotolerance of the *S. cerevisiae* parent (Bellon *et al*., 2011, 2013). When *S. cerevisiae* was hybridized with *S. eubayanus*, the allotriploids and the allotetraplois, but not the allodiploids, outperformed the parental strains in fermentation of very high‐gravity wort (Krogerus *et al*., 2016).

## Postzygotic genome segregation and chimerization broadens phenotypic diversity

### Postzygotic shaping of the hybrid genome (postzygotic genome evolution)

The hybrid genomes are usually unstable and prone to ‘postzygotic’ changes either during vegetative (mitotic) propagation of the sterile alloploid cells or during sporulation of the allopolyploid cells upon the breakdown of their sterility. As both processes are associated with spontaneous loss of chromosomes, the terms GARMi (Genome AutoReduction in Mitosis) and GARMe (Genome AutoReduction in Meiosis) were recently proposed to designate them (Sipiczki, 2018) (Fig. 2B).

In GARMi (usually referred to as ‘hybrid stabilization’ or ‘hybrid evolution’), recurrent unequal mitotic divisions (biased segregation) generate alloaneuploid cells lacking chromosomes or arms of chromosomes in one or the other subgenome (for a review, see Sipiczki, 2018). GARMe can take place after the breakdown of the sterility barrier in allotetraploid meiosis by the loss of *MAT* heterozygosity (malsegregation of the *MAT*‐carrying chromosomes in one of the subgenomes) (Pfliegler *et al*., 2012; Karanyicz *et al*., 2017). The alloaneuploid spores establish vegetatively propagating clones of mating‐competent cells (F1 spore clones, ‘propagating gametes’) which can conjugate with each other (selfing) to form sporulation‐proficient alloaneuploid zygotes producing alloaneuploid cells (F2 generation). As aneuploidy usually destabilizes the genome, the F2 cells can easily lose additional chromosomes when sporulate. The outcomes of both processes are strains of chimeric (mosaic) genomes composed of various combinations and proportions of the parental gene pools.

### Postzygotic genomic changes are associated with novel phenotypic traits

Several studies have shown that the prolonged cultivation of a hybrid under a specific stress condition (high ethanol or sugar concentration, nutrient‐limited conditions, extreme temperature, etc.) gradually improves its fitness (e.g. Piotrowski *et al*., 2012; Dunn *et al*., 2013; Lopandic *et al*., 2016; Smukowski Heil *et al*., 2017; Krogerus *et al*., 2018 **)**. Where investigated, the improved fitness turned out to be associated with the accumulation of segregants (‘evolved hybrids’ or ‘evolved variants’) of specific types of chimeric genomes in the population. The genetic changes are most probably due to spontaneous missegregation and/or structural changes of certain chromosomes at mitotic divisions, and to randomly occurring mutations. The stress factor does not induce these genetic changes; it only differentially affects the growth of the derivatives if they differ in fitness. The less sensitive derivatives will gradually overgrow the rest of the population. Different conditions lead to different genomic outcomes from the same hybrid (e.g. Piotrowski *et al*., 2012; Lopandic *et al*., 2016).

The gradual enrichment of the hybrid culture with a specific derived segregant is nicely illustrated by the results of Bellon *et al*. (2018) who let a *S. cerevisiae* × *S. uvarum* hybrid propagate vegetatively through a series of successive fermentations of a botrytized Riesling must of high sugar content (350 g l^−1^ initial sugar) and monitored the changes in the population. After the 5th round of fermentation, 95% of the cells lacked the entire chromosome 14 of *S. uvarum* and the rest lacked its left arm. It was hypothesized that the loss of the *S. uvarum* chromosome 14 accounted for the increased fitness of the population. However, chromosome 14 instability had been observed also in *S. cerevisiae* × *S. uvarum* hybrids exposed to nitrogen limiting conditions (Dunn *et al*., 2013). Thus, the loss of this chromosome may not be specific for the high‐sugar stress response.

In a different study (Lopandic *et al*., 2016), no karyotype changes (no GARMi) were detected in *S. cerevisiae* × *S*. *uvarum* and *S. cerevisiae* × *S. kudriavzevii* hybrids after the fermentation of a high‐sugar grape must (Grüner Veltliner with an initial sugar concentration of 337.5 g l^−1^) but both hybrids segregated at meiosis (GARMe). As the spores were viable (produced colonies), the hybrids must have been allotetraploid. The tetrad isolated from the *S. cerevisiae* × *S. uvarum* hybrid showed the segregation pattern characteristic of the tetrads in which the (second) sterility barrier breaks down (Pfliegler *et al*., 2012): two spores lacked the *MAT*‐carrying chromosome of the *S. uvarum* subgenome. The clones established by these alloaneuploid spores remained stable during fermentation, whereas the other two, presumably allodiploid spore clones underwent GARMi, resulting in mixed populations of cells lacking various chromosomes mostly in the *S. uvarum* subgenome. In contrast to the finding of the Bellon laboratory (Bellon *et al*., 2018), no chromosome 14 instability was detected here. The spore clones that retained the *MAT*‐carrying chromosomes of both parents showed the phenotype of the hybrid: had higher fermentation rates and richer aroma profile in the high‐glucose must, produced more ethanol and less volatile acids than the other spores and the parental strains (Lopandic *et al*., 2016; Gangl *et al*., 2017). The karyotype of the *S. cerevisiae* × *S. kudriavzevii* hybrid also showed 2:2 segregation (GARMe), but different chromosomes were involved, and all spore clones performed worse than the hybrid for all parameters examined. The segregation of AFLP (amplified fragment length polymorphism) pattern in these alloaneuploids was much more complex, indicating that smaller‐scale GARMe, and GARMi changes took place both within the subgenomes and between them. The observed diversity of spore clones indicates that meiotic segregation poses a risk to the application of hybrids of genomes larger than allodiploid in fermentation technologies.

## New perspectives in three‐species hybridizations

**Figure 3:**
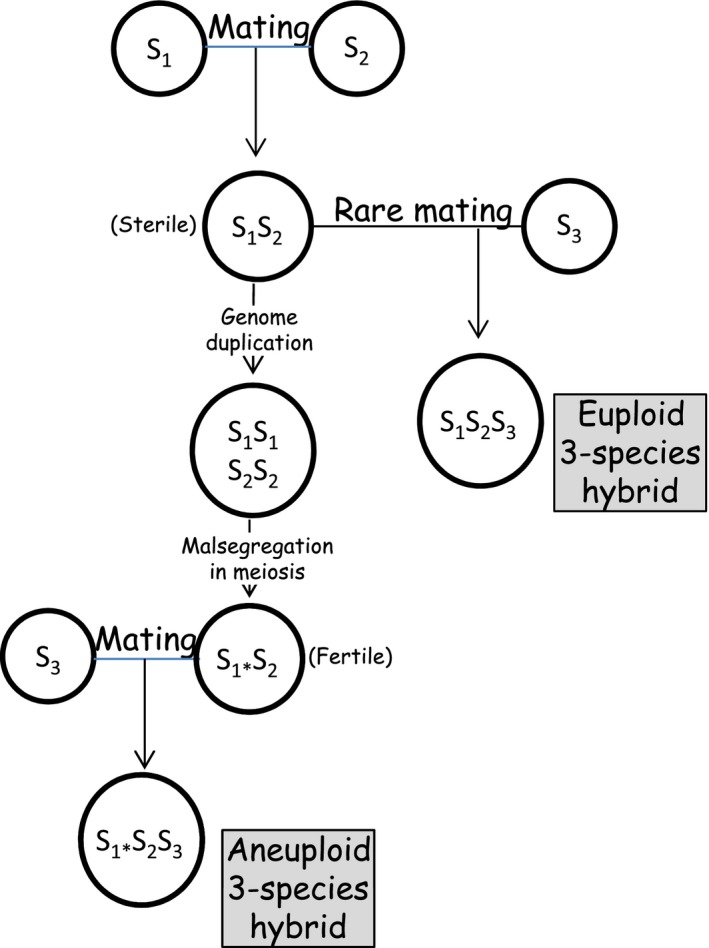
Strategies of three‐species hybridization. S_1_, S_2_, and S_3_: different *Saccharomyces* species. Star marks incomplete subgenome.

The gene pools of the two‐species *Saccharomyces* hybrids or their alloaneuploid or chimeric derivatives can be enriched with genes from a third species by mating with a strain of the third species (e.g. Sipiczki *et al*., 2014; Antunovics *et al*., 2018a,2018b). Two strategies have been developed for the construction of three‐species synthetic hybrids and/or genomic chimeras (Antunovics *et al*., 2018b) (Fig. 3). One method is based on the fertility of the alloaneuploid spore clones produced in GARMe after the breakdown of the sterility barrier in tetraploid meiosis. These clones sporulate, and their spores can be mass‐mated with spores of a homothallic strain or with cells of a heterothallic strain of the third species. The outcome of the process is a chimeric strain with a complete genome of the third species and incomplete genomes of the parental strains of the two‐species alloaneuploid. As spores of diverse alloaneuploid/chimeric genomes are produced during GARMe of the two‐species hybrid, the arising zygotes will have diverse genomes composed of various proportions of the genes of the three parental species. The other approach generates true allotriploid hybrids having complete genomes of all three species. Their construction starts with hybridizing two species and selecting a stabile (sterile) allodiploid hybrid. This sterile *MATa/MATalpha* hybrid is then mated with fertile spores or cells of the third species by taking advantage of the phenomenon referred to as ‘rare mating’ (Gunge and Nakatomi, 1972). *MAT* heterozygosity suppresses the mating‐specific genes and makes the allodiploid cells mating‐incompetent, but very rarely certain cells escape the block and conjugate with mating‐competent cells or spores of a different strain. *S. cerevisiae* × *S. kudriavzevii* × *S. uvarum* (‘cekudvarum’) chimeric and allotriploid hybrid strains have been recently produced with these methods (Antunovics *et al*., 2018a,2018b). Both types of three‐species strains showed transgressive phenotypic traits. Although their response to high‐sugar stress has not been examined, improved osmotolerance can be expected in certain strains or in their GARMI/GARME derivatives because natural isolates of composite genomes consisting of genes from these species had phenotypes suitable to overcome stuck fermentation (Christ *et al*., 2015).

## Conflict of interest

None declared.

## References

[mbt213390-bib-0001] Albertyn, J. , Hohmann, S. , Thevelein, J.M. , and Prior, B.A. (1994) *GPD1*, which encodes glycerol‐3‐phosphate dehydrogenase, is essential for growth under osmotic stress in *Saccharomyces cerevisiae*, and its expression is regulated by the high‐osmolarity glycerol response pathway. Mol Cell Biol 14: 4135–4144. 8196651 10.1128/mcb.14.6.4135-4144.1994 PMC358779

[mbt213390-bib-0002] Ando, A. , Tanaka, F. , Murata, Y. , Takagi, H. , and Shima, J. (2006) Identification and classification of genes required for tolerance to high‐sucrose stress revealed by genome‐wide screening of *Saccharomyces cerevisiae* . FEMS Yeast Res 6: 249–267. 16487347 10.1111/j.1567-1364.2006.00035.x

[mbt213390-bib-0003] Antunovics, Z. , Czentye, K. , Hostyisoczky, A. , and Sipiczki, M. (2018a) Molecular genetic analysis of synthetic *Saccharomyces cerevisiae* x *S. kudriavzevii* x *S. uvarum* three‐species hybrids with emphasis on the inheritance of mitochondria In 45th Annual Conference on Yeasts. Hapala I., and Certik M. (eds). Bratislava, Slovakia: Czechoslovak Society for Microbiology, p. 39.

[mbt213390-bib-0004] Antunovics, Z. , Karanyicz, E. , Szabo, A. , Pfliegler, W.P. and Sipiczki, M. (2018b) Synthetic two‐ and three‐species hybridisation in *Saccharomyces*: non‐introgressive genome chimerisation and mitochondrial inheritance In 34th International Specialised Symposium on Yeasts. Libkind D. (ed). Bariloche, Argentina: International Commission on Yeasts, p. 128.

[mbt213390-bib-0005] Auesukaree, C. (2017) Molecular mechanisms of the yeast adaptive response and tolerance to stresses encountered during ethanol fermentation. J Biosci Bioeng 124: 133–142. 28427825 10.1016/j.jbiosc.2017.03.009

[mbt213390-bib-0006] Bellon, J.R. , Eglinton, J.M. , Siebert, T.E. , Pollnitz, A.P. , Rose, L. , de Barros Lopes, M. , *et al* (2011) Newly generated interspecific wine yeast hybrids introduce flavor and aroma diversity to wines. Appl Microbiol Biotechnol 91: 603–612. 21538112 10.1007/s00253-011-3294-3

[mbt213390-bib-0007] Bellon, J.R. , Schmid, F. , Capone, D.L. , Dunn, B.L. , and Chambers, P.J. (2013) Introducing a new breed of wine yeast: interspecific hybridization between a commercial *Saccharomyces cerevisiae* wine yeast and *Saccharomyces mikatae* . PLoS ONE 8: e62053. 23614011 10.1371/journal.pone.0062053 PMC3629166

[mbt213390-bib-0008] Bellon, J.R. , Yang, F. , Day, M.P. , Inglis, D.L. , and Chambers, P.J. (2015) Designing and creating *Saccharomyces* interspecific hybrids for improved, industry relevant, phenotypes. Appl Microbiol Biotechnol 99: 8597–8609. 26099331 10.1007/s00253-015-6737-4

[mbt213390-bib-0009] Bellon, J.R. , Ford, C.M. , Borneman, A.R. , and Chambers, P.J. (2018) A novel approach to isolating improved industrial interspecific wine yeasts using chromosomal mutations as potential markers for increased fitness. Front Microbiol 9: 1442. 30034376 10.3389/fmicb.2018.01442 PMC6043810

[mbt213390-bib-0010] Chowdhury, S. , Smith, K.W. , and Gustin, M.C. (1992) Osmotic stress and the yeast cytoskeleton: phenotype‐specific suppression of an actin mutation. J Cell Biol 118: 561–571. 1639843 10.1083/jcb.118.3.561 PMC2289551

[mbt213390-bib-0011] Christ, E. , Kowalczyk, M. , Zuchowska, M. , Claus, H. , Löwenstein, R. , Szopinska‐Morawska, A. , *et al* (2015) An exemplary model study for overcoming stuck fermentation during spontaneous fermentation with the aid of a *Saccharomyces* triple hybrid. J Agric Sci 7: 18–34.

[mbt213390-bib-0012] Doelle, M.B. , and Docile, H.W. (1990) Sugar‐cane molasses fermentation by *Zymomonas mobilis* . Appl Microbiol Biotechnol 33: 31–35.

[mbt213390-bib-0013] Donéche, B.J. (1993) Botrytized wines In Wine Microbiology and Biotechnology. Fleet G.H. (ed). Chur, Switzerland: Harwood Academic Publishers, pp. 327–351.

[mbt213390-bib-0014] Dunn, B.L. , Paulish, T. , Stanbery, A. , Piotrowski, J. , Koniges, G. , Kroll, E. , *et al* (2013) Recurrent rearrangement during adaptive evolution in an interspecific yeast hybrid suggests a model for rapid introgression. PLoS Genet 9: e1003366. 23555283 10.1371/journal.pgen.1003366 PMC3605161

[mbt213390-bib-0015] Erasmus, D.J. , van der Merwe, G.K. , and van Vuuren, H.J.J. (2003) Genome‐wide expression analyses: metabolic adaptation of *Saccharomyces cerevisiae* to high sugar stress. FEMS Yeast Res 3: 375–399. 12748050 10.1016/S1567-1356(02)00203-9

[mbt213390-bib-0016] Erasmus, D.J. , Cliff, M. , and van Vuuren, H.J.J. (2004) Impact of yeast strain on the production of acetic acid, glycerol, and the sensory attributes of icewines. Am J Enol Vitic 55: 371–378.

[mbt213390-bib-0017] Erny, C. , Raoult, P. , Alais, A. , Butterlin, G. , Delobel, P. , Matei‐Radoi, F. , *et al* (2012) Ecological success of group of *Saccharomyces cerevisiae*/*Saccharomyces kudriavzevii* hybrids in the northern European wine‐making environment. Appl Environ Microbiol 78: 3256–3265. 22344648 10.1128/AEM.06752-11 PMC3346444

[mbt213390-bib-0018] Gangl, H. , Tiefenbrunner, W. , Pfliegler, W.P. , Sipiczki, M. , Leitner, G. , Tscheik, G. , and Lopandic, K. (2017) Influence of artificial interspecies yeast hybrids and their F1 offspring on the aroma profile of wine. Mitt Klosterneuburg 67: 68–83.

[mbt213390-bib-0019] Garcia Sanchez, R. , Solodovnikova, N. , and Wendland, J. (2012) Breeding of lager yeast with *Saccharomyces cerevisiae* improves stress resistance and fermentation performance. Yeast 29: 343–355. 22887121 10.1002/yea.2914

[mbt213390-bib-0020] Gonzalez, R. , Morales, P. , Tronchoni, J. , Cordero‐Bueso, G. , Vaudano, E. , Quirós, M. , *et al* (2016) New genes involved in osmotic stress tolerance in *Saccharomyces cerevisiae* . Front Microbiol 7: 1545. 27733850 10.3389/fmicb.2016.01545 PMC5039201

[mbt213390-bib-0021] Gunge, N. , and Nakatomi, Y. (1972) Genetic mechanisms of rare matings of the yeast *Saccharomyces cerevisiae* heterozygous for mating type. Genetics 70: 41–58. 17248555 10.1093/genetics/70.1.41 PMC1212722

[mbt213390-bib-0022] Hohmann, S. (2015) An integrated view on a eukaryotic osmoregulation system. Curr Genet 61: 373–382. 25663258 10.1007/s00294-015-0475-0

[mbt213390-bib-0023] Huuskonen, A. , Markkula, T. , Vidgren, V. , Lima, L. , Mulder, L. , Geurts, W. , *et al* (2010) Selection from industrial lager yeast strains of variants with improved fermentation performance in very‐high‐gravity worts. Appl Environ Microbiol 76: 1563–1573. 20081007 10.1128/AEM.03153-09 PMC2832358

[mbt213390-bib-0024] Jimenez‐Marti, E. , Gomar‐Alba, M. , Palacios, A. , Ortiz‐Julien, A. , and del Olmo, M. (2011) Towards an understanding of the adaptation of wine yeasts to must: relevance of the osmotic stress response. Appl Microbiol Biotechnol 89: 1551–1561. 20941492 10.1007/s00253-010-2909-4

[mbt213390-bib-0025] Karanyicz, E. , Antunovics, Z. , Kallai, Z. , and Sipiczki, M. (2017) Non‐introgressive genome chimerisation by malsegregation in autodiploidised allotetraploids during meiosis of *Saccharomyces kudriavzevii* x *Saccharomyces uvarum* hybrids. Appl Microbiol Biotechnol 101: 4617–4633. 28396924 10.1007/s00253-017-8274-9

[mbt213390-bib-0026] Khoja, A.H. , Yahya, S.M. , Nawar, A. , Ansari, A.A. , and Qayyum, M. (2018) Fermentation of sugarcane molasses using *Zymomonas mobilis* for enhanced bioethanol production. J Adv Res Appl Sci Eng Technol 11: 31–38.

[mbt213390-bib-0027] Krogerus, K. , Arvas, M. , De Chiara, M. , Magalhães, F. , Mattinen, L. , Oja, M. , *et al* (2016) Ploidy influences the functional attributes of de novo lager yeast hybrids. Appl Microbiol Biotechnol 100: 7203–7222. 27183995 10.1007/s00253-016-7588-3 PMC4947488

[mbt213390-bib-0028] Krogerus, K. , Holmström, S. , and Gibson, B. (2018) Enhanced wort fermentation with *De Novo* lager hybrids adapted to high‐ethanol environments. App Environ Microbiol 84: e02302–e02317. 10.1128/AEM.02302-17 PMC5795086 29196294

[mbt213390-bib-0030] Legmann, R. , and Margalith, P. (1983) Interspecific protoplast fusion of *S. cerevisiae* and *S. mellis* . Eur J Appl Microbiol Biotechnol 18: 320–322.

[mbt213390-bib-0031] Legmann, R. , and Margalith, P. (1986) Ethanol formation by hybrid yeasts. Eur J Appl Microbiol Biotechnol 23: 198–202.

[mbt213390-bib-0032] Lopandic, K. , Pfliegler, W. , Tiefenbrunner, W. , Gangl, H. , Sipiczki, M. , and Sterflinger, K. (2016) Genotypic and phenotypic evolution of yeast interspecies hybrids during high‐sugar fermentation. Appl Microbiol Biotechnol 100: 6331–6343. 27075738 10.1007/s00253-016-7481-0

[mbt213390-bib-0033] Loray, M.A. , Spencer, J.F.T. , Spencer, D.M. , and de Figueroa, L.I.C. (1995) Hybrids obtained by protoplast fusion with a salt‐tolerant yeast. J Ind Microbiol 14: 508–513. 7662292 10.1007/BF01573966

[mbt213390-bib-0034] Lucca, M.E. , Loray, M.A. , de Figueroa, L.I.C. , and Callieri, D.A.S. (1999) Characterization of osmotolerant hybrids obtained by fusion between protoplasts of *Saccharomyces cerevisiae* and heat treated protoplasts of *Torulaspora delbrueckii* . Biotechnol Lett 21: 343–348.

[mbt213390-bib-0035] Lucca, M.E. , Spencer, J.F. , and de Figueroa, L.I. (2002) Glycerol and arabitol production by an intergeneric hybrid, PB2, obtained by protoplast fusion between *Saccharomyces cerevisiae* and *Torulaspora delbrueckii* . Appl Microbiol Biotechnol 59: 472–476. 12172612 10.1007/s00253-002-1025-5

[mbt213390-bib-0500] Marsit, S. , and Dequin, S. (2015) Diversity and adaptive evolution of Saccharomyces wine yeast: a review. FEMS Yeast Res 15: pii: fov067. 10.1093/femsyr/fov067 PMC4629790 26205244

[mbt213390-bib-0037] Munna, M.S. , Humayun, S. , and Noor, R. (2015) Influence of heat shock and osmotic stresses on the growth and viability of *Saccharomyces cerevisiae* SUBSC01. BMC Res Notes 8: 369. 26298101 10.1186/s13104-015-1355-x PMC4546815

[mbt213390-bib-0038] Ok, T. , and Hashinaga, F. (1997) Identification of sugar‐tolerant yeasts isolated from high‐sugar fermented vegetable extracts. J Gen Appl Microbiol 43: 39–47. 12501352 10.2323/jgam.43.39

[mbt213390-bib-0039] O'Rourke, S.M. , and Herskowitz, I. (2004) Unique and redundant roles for HOG MAPK pathway components as revealed by whole‐genome expression analysis. Mol Biol Cell 15: 532–542. 14595107 10.1091/mbc.E03-07-0521 PMC329229

[mbt213390-bib-0040] Pastor, M.M. , Proft, M. , and Pascual‐Ahuir, A. (2009) Mitochondrial function is an inducible determinant of osmotic stress adaptation in yeast. J Biol Chem 284: 30307–30317. 19720830 10.1074/jbc.M109.050682 PMC2781586

[mbt213390-bib-0041] Peris, D. , Pérez‐Torrado, R. , Hittinger, C.T. , Barrio, E. , and Querol, A. (2018) On the origins and industrial applications of *Saccharomyces cerevisiae* × *Saccharomyces kudriavzevii* hybrids. Yeast 35: 51–69. 29027262 10.1002/yea.3283

[mbt213390-bib-0042] Pfliegler, W.P. , Antunovics, Z. , and Sipiczki, M. (2012) Double sterility barrier between *Saccharomyces* species and its breakdown in allopolyploid hybrids by chromosome loss. FEMS Yeast Res 12: 703–718. 22697168 10.1111/j.1567-1364.2012.00820.x

[mbt213390-bib-0043] Pfliegler, W.P. , Atanasova, A. , Karanyicz, E. , Sipiczki, M. , Bond, U. , Druzhinina, I.S. , *et al* (2014) Generation of new genotypic and phenotypic features in artificial and natural yeast hybrids. Food Technol Biotechnol 52: 46–57.

[mbt213390-bib-0044] Piotrowski, J.S. , Nagarajan, S. , Kroll, E. , Stanbery, A. , Chiotti, K.E. , Kruckeberg, A.L. , *et al* (2012) Different selective pressures lead to different genomic outcomes as newly‐formed hybrid yeasts evolve. BMC Evol Biol 12: 46. 22471618 10.1186/1471-2148-12-46 PMC3372441

[mbt213390-bib-0045] Pratt, P.L. , Bryce, J.H. , and Stewart, G.G. (2003) The effects of osmotic pressure and ethanol on yeast viability and morphology. J Inst Brew 109: 218–228.

[mbt213390-bib-0047] Restuccia, C. , Muratore, G. , Muccilli, S. , Randazzo, C.L. , Caggia, C. , Mazzaglia, A. , *et al* (2011) *Saccharomyces* hybrids as a tool for improving the quality of moscato Di siracusa DOC wine. Ital J Food Sci 23: 28–35.

[mbt213390-bib-0048] Saito, H. , and Posas, F. (2012) Response to hyperosmotic stress. Genetics 192: 289–318. 23028184 10.1534/genetics.112.140863 PMC3454867

[mbt213390-bib-0049] Salek, A.T. (2002) Classic techniques for improvement of industrial yeast strains. Biotechnologia 1: 153–174.

[mbt213390-bib-0051] Sasano, Y. , Haitani, Y. , Ohtsu, I. , Shima, J. , and Takagi, H. (2012) Proline accumulation in baker's yeast enhances high‐sucrose stress tolerance and fermentation ability in sweet dough. Int J Food Microbiol 152: 40–43. 22041027 10.1016/j.ijfoodmicro.2011.10.004

[mbt213390-bib-0052] Saxena, A. , and Sitaraman, R. (2016) Osmoregulation in *Saccharomyces cerevisiae* via mechanisms other than the high‐osmolarity glycerol pathway. Microbiology 162: 1511–1526. 27557593 10.1099/mic.0.000360

[mbt213390-bib-0053] Schaber, J. , and Klipp, E. (2008) Short‐term volume and turgor regulation in yeast. Essays Biochem 45: 147–159. 18793130 10.1042/BSE0450147

[mbt213390-bib-0054] Shi, X. , Zou, Y. , Chen, Y. , and Ying, H. (2018) Overexpression of *THI4* and *HAP4* improves glucose metabolism and ethanol production in *Saccharomyces cerevisiae* . Front Microbiol 9: 1444. 29997610 10.3389/fmicb.2018.01444 PMC6030257

[mbt213390-bib-0055] Sipiczki, M. (2018) Interspecies hybridisation and genome chimerisation in *Saccharomyces*: combining of gene pools of species and its biotechnological perspectives. Front Microbiol 9: 3071. 30619156 10.3389/fmicb.2018.03071 PMC6297871

[mbt213390-bib-0056] Sipiczki, M. , Csoma, H. , Antunovics, Z. , and Pfliegler, W.P. (2010) Biodiversity in yeast populations associated with botrytised wine making. Mitt Klosterneuburg 60: 387–394.

[mbt213390-bib-0057] Sipiczki, M. , Pfliegler, W.P. , Karanyicz, E. , Antunovics, Z. , and Kallai, Z. (2014) Combined gene pools of *Saccharomyces* species: production and oenological potentials of *cevarum*,* kudvarum* and *cekudvarum* mosaic genomes In 37th World Congress of Vine and Wine. Aurand J.‐M. (ed). Mendoza, Argentina: International Organisation of Vine and Wine, pp. 554–555.

[mbt213390-bib-0058] Slaninova, I. , Sestak, S. , Svoboda, A. , and Farkas, V. (2000) Cell wall and cytoskeleton reorganization as the response to hyperosmotic shock in *Saccharomyces cerevisiae* . Arch Microbiol 173: 245–252. 10816042 10.1007/s002030000136

[mbt213390-bib-0059] Smukowski Heil, C.S. , DeSevo, G.C. , Pai, D.A. , Tucker, C.M. , Hoanh, M.L. , and Dunham, M.J. (2017) Loss of heterozygosity drives adaptation in hybrid yeast. Mol Biol Evol 34: 1596–1612. 28369610 10.1093/molbev/msx098 PMC5455960

[mbt213390-bib-0060] Spencer, J.F.T. , Bizeau, C. , Reynolds, N. , and Spencer, D.M. (1985) The use of mitochondrial mutants in hybridization of industrial yeast strains. VI. Characterization of the hybrid, *Saccharomyces diastaticus* x *Saccharomyces rouxii*, obtained by protoplast fusion, and its behavior in simulated dough‐raising tests. Curr Genet 9: 649–652.

[mbt213390-bib-0061] Stewart, G.G. , Russell, I. , and Panchal, C.J. (1988) Genetically stable allopolyploid somatic fusion product useful in the production of fuel alcohols. United States Patent Patent Number: 4,772,556.

[mbt213390-bib-0062] Talemi, S.R. , Tiger, C.F. , Andersson, M. , Babazadeh, R. , Welkenhuysen, N. , Klipp, E. , *et al* (2016) Systems level analysis of the yeast osmo‐stat. Sci Rep 6: 30950. 27515486 10.1038/srep30950 PMC4981887

[mbt213390-bib-0063] Tanaka‐Tsuno, F. , Mizukami‐Murata, S. , Murata, Y. , Nakamura, T. , Ando, A. , Takagi, H. , and Shima, J. (2007) Functional genomics of commercial baker's yeasts that have different abilities for sugar utilization and high‐sucrose tolerance under different sugar conditions. Yeast 24: 901–911. 17724779 10.1002/yea.1541

[mbt213390-bib-0064] Taymaz‐Nikerel, H. , Cankorur‐Cetinkaya, A. , and Kirdar, B. (2016) Genome‐wide transcriptional response of *Saccharomyces cerevisiae* to stress‐induced perturbations. Front Bioeng Biotechnol 4: 17. 26925399 10.3389/fbioe.2016.00017 PMC4757645

[mbt213390-bib-0065] Watanabe, T. , Srichuwong, S. , Arakane, M. , Tamiya, S. , Yoshinaga, M. , Yamamoto, M. , *et al* (2010) Selection of stress‐tolerant yeasts for simultaneous saccharification and fermentation (SSF) of very high gravity (VHG) potato mash to ethanol. Bioresour Technol 101: 9710–9714. 20705456 10.1016/j.biortech.2010.07.079

